# Environmental factors and risk of delirium in geriatric patients: an observational study

**DOI:** 10.1186/s12877-018-0977-y

**Published:** 2018-11-15

**Authors:** Sigurd Evensen, Ingvild Saltvedt, Stian Lydersen, Torgeir Bruun Wyller, Kristin Taraldsen, Olav Sletvold

**Affiliations:** 1 Department of Geriatrics, St. Olavs hospital, Trondheim University Hospital, Trondheim, Norway; 2 Department of Neuromedicine and Movement Science, Faculty of Medicine and Health Sciences, Norwegian University of Science and Technology (NTNU), N-7491 Trondheim, Norway; 3 Regional Centre for Child and Youth Mental Health and Child Welfare, NTNU, Norwegian University of Science and Technology, Trondheim, Norway; 4 Oslo Delirium Research Group, Department of Geriatric Medicine, Oslo University Hospital, Oslo, Norway; 5 Institute of Clinical Medicine, University of Oslo, Oslo, Norway

**Keywords:** Delirium, Risk factors, Geriatric patients, Hospital, Environmental

## Abstract

**Background:**

Patients with delirium have increased risk of death, dementia and institutionalization, and prognosis differs between delirium motor subtypes. A few studies have identified associations between environmental factors like room-transfers and time spent in the emergency department (ED) and delirium, but no studies have investigated if environmental factors may influence delirium motor subtypes. We wanted to explore if potentially stressful events like ward-transfers, arriving ED at nighttime, time spent in ED and nigthttime investigations were associated with development of delirium (incident delirium) and delirium motor subtypes.

**Methods:**

We used the DSM-5 criteria to diagnose delirium and the Delirium Motor Subtype Scale for motor subtyping. We defined hyperactive and mixed delirium as delirium with hyperactive symptoms, and hypoactive and no-subtype delirium as delirium without hyperactive symptoms. We registered ward-transfers, time of arrival in ED, time spent in ED and nighttime investigations (8 p.m. to 8 a.m.), and calculated Global Deterioration Scale (GDS) and Cumulative Illness Rating Scale (CIRS) to adjust for cognitive impairment and comorbidity. We used logistic regression analyses with incident delirium and delirium with hyperactive symptoms as outcome variables, and ward-transfers, arriving ED at nighttime, time spent in ED and nighttime investigations as exposure variables, adjusting for age, GDS and CIRS in the analyses for incident delirium.

**Results:**

We included 254 patients, mean age 86.1 years (SD 5.2), 49 (19.3%) had incident delirium, 22 with and 27 without hyperactive symptoms. There was a significant association between nighttime investigations and incident delirium in both the unadjusted (odds ratio (OR) 2.22, 95% confidence interval (CI) 1.17 to 4.22, *p* = 0.015) and the multiadjusted model (OR 2.61, CI 1.26 to 5.40, *p* = 0.010). There were no associations between any other exposure variables and incident delirium. No exposure variables were associated with delirium motor subtypes.

**Conclusions:**

Nighttime investigations were associated with incident delirium, even after adjusting for age, cognitive impairment and comorbidity. We cannot out rule that the medical condition leading to nighttime investigations is the true delirium-trigger, so geriatric patients must still receive emergency investigations at nighttime. Hospital environment in broad sense may be a target for delirium prevention.

## Background

Delirium is an acute disturbance of attention, awareness and cognition, affecting one third of older medical inpatients [1]. High age, cognitive impairment and comorbidity are the most important risk factors [2]. Patients suffering from delirium have increased risk of death, cognitive impairment and institutionalization [3], and delirium has substantial medical, societal and economical implications on the entire health care system [4]. Four different motor subtypes of delirium have been described; hyperactive delirium, hypoactive delirium, mixed delirium with both hyperactive and hypoactive features and no-subtype delirium without motor disturbances [5]. Most studies find that hypoactive delirium has worst prognosis [5–9]. It is unclear whether risk factors and etiology differ between motor subtypes [8, 10, 11].

The Diagnostic and Statistical Manual of Mental Disorders (DSM-5) criteria [12] are physiologically oriented and state that delirium is caused by medical conditions, substance intoxication or withdrawal, exposure to a toxin, or is due to multiple etiologies. On the other hand, non-pharmacological intervention programs focusing on activity, orientation and sleep hygiene are effective to prevent delirium [13, 14], indicating that environmental factors may have a role in development of delirium. There are previous reports on both sensory deprivation [15] and sensory overload [16] as contributors to delirium, and three studies have identified associations between specific environmental factors in the hospital care pathway and delirium. Goldberg et al. and Bo et al. found associations between room-transfers and time spent in the emergency department (ED) and development of delirium, respectively [17, 18], and McCusker et al. found that increasing number of room-transfers increased the severity of delirium [15]. These associations seem plausible since both room-transfers and long time spent in ED can be stressfull events that might be able to induce aberrant stress responses eventually contributing to delirium [19]. To our knowledge, no studies have investigated the association between environmental factors and motor subtypes of delirium, which is of interest since the motor subtypes have different prognosis.

Due to the substantial impact of delirium and the increasing number of delirium-prone older patients in strained and crowded hospitals [20], there is a need to further explore the associations between potentially stressful environmental factors in the hospital care patway and delirium. The aim of this study is to specifically investigate if ward-transfers, arriving ED at nighttime, time spent in ED and visits from other specialists and radiological procedures at nighttime (nighttime investigations) are associated with development of delirium (incident delirium) and delirium motor subtypes in patients acutely admitted to a geriatric ward.

## Methods

### Design, settings and participants

This is a prospective observational study conducted at the medical geriatric ward at St. Olavs hospital, Trondheim University Hospital, Norway, between May 6 2015 and January 31 2017. The ward has 15 single-bed rooms, and the patients receive comprehensive geriatric assessment and care [21] by an interdisciplinary geriatric team consisting of physicians, nurses, occupational therapists and physiotherapists. Ninety per cent of the patients are acutely admitted with conditions like infections, injuries after falls, cardiopulmonary conditions and dehydration [22]. Acutely admitted patients arrive via the ED which has ten regular rooms, three acute-rooms and eight beds in a triage room. Nurses collect blood-samples in the ED. Physicians examine the patients in the ED before the patients as soon as possible are transferred to a relevant ward. The patients frequently receive radiological procedures during transfer between ED and the ward. As in other hospitals [20], the ED is frequently chaotic and over-crowded.

The inclusion criteria were age ≥ 75 years and acute admittance. Patients transferred from other wards were eligible for inclusion if they met the inclusion criteria. We excluded patients previously taking part in the study and patients with delirium on admittance. Nurses, physiotherapists or a physician (SE) included all patients within 24 h after arriving the ward.

### Diagnosis of delirium and delirium motor subtypes

We diagnosed delirium according to the DSM-5 criteria, judging consciousness, awareness and arousal clinically, testing attention using the digit span forwards and backwards [23] and cognitive impairment using the ten orientation items and the three word short time memory test from the Mini Mental Status Excamination [24]. In this population of elderly patients we particularily stressed that the present symptoms could not be better explained by preexisting dementia and that the delirium episode had to be a consequence of physiological disturbances. We based the final diagnosis on all available information, i.e. first day visits to all patients, interviews with nurses and proxies and careful chart review as described by Inouye [25], since this combined approach increases the number of patients correctly diagnosed with delirium [26]. When in doubt concerning the diagnosis, and if the staff noticed changes in mental status, we visited the patient several times.

After diagnosing delirium, we did motor subtyping using the Delirium Motor Subtype Scale (DMSS) [27]. The DMSS lists four hyperactive and seven hypoactive features. To fulfill the criteria for a certain motor subtype, the patient must have at least two of these features. Patients having both hyperactive and hypoactive features get the diagnosis of mixed delirium, and patients with one or less motor feature get the diagnosis of no-subtype delirium. Due to a small number of observations, we combined the patients with hyperactive and mixed delirium to create the category “delirium with hyperactive symptoms” and the patients with hypoactive and no-subtype delirium to create the category “delirium without hyperactive symptoms.” In patients not visited due to logistical reasons, we based the diagnosis of delirium and motor subtype on careful chart review. We were not able to secure that the delirium assessors were completely blinded to exposure status of environmental factors.

### Data collection

We registered time of arrival at the ED and total time in the ED retrospectively using the hospital’s ED database. We defined nighttime between 8 p.m. and 8 a.m. We registered cerebral MRI-scans, other radiological investigations (CT-scans, ultrasound and x-rays) and visits from other specialties at nighttime retrospectively reviewing the hospital records. Due to small numbers of cerebral MRI-scans and visits from other specialties we combined these with other radiological investigations and created the category investigations at nighttime, despite that MRI-scans might be a stronger contributor to incident delirium due to noise and narrowness. Nurses registered ward-transfers consecutively (yes/no).

To be able to adjust for cognitive impairment, we scored the Global Deterioration Scale (GDS) [28]. The GDS ranges from one to seven, one indicates no cognitive symptoms, seven indicates end-stage dementia. We defined dementia as a GDS-score more than four. To be able to adjust for comorbidity, we calculated the Cumulative Illness Rating Scale (CIRS) retrospectively reviewing the hospital records [29]. CIRS ranges from 0 to 56; higher score indicates increasing comorbidity.

We used the Barthel Index (BI, 0 to 20, 20 best score) as a baseline measure of personal Activities of Daily Living [30] prior to hospitalization and the Short Physical Performance Battery (SPPB, 0 to 12, 12 best score) as a baseline measure of general health and frailty [31]. We used a modified APACHE II-score (0 to 71, increasing score indicates more severe illness) as a baseline measure of acute illness [32]. We collected demographic data from the hospital records.

### Ethics

The Regional Committee for Medical and Health Research Ethics of Mid-Norway approved the study (REC Central 2015/474). Since there were no elements of invasive or uncomfortable procedures, the patients could consent for participation even if they had signs of cognitive impairment. If the patient was unable to give consent, a proxy could sign the consent form. Independent of cognitive status, we never included patients who expressed concerns about participation.

### Statistical analysis

We present descriptive data for continuous variables as means and standard deviations (SD), and for dichotomous and categorical variables as percentages. To investigate if the exposure variables ward-transfers, arrival at nighttime, time spent in ED and nighttime investigations were associated with incident delirium, we used logistic regression analyses, unadjusted and multiadjusted, with incident delirium as outcome variable. To adjust for important risk factors for delirium we also included the covariates age, GDS (cognitive function) and CIRS (comorbidity) in the analyses. We used the same strategy to study the relation between the exposure variables and motor subtypes, using incident delirium with hyperactive symptoms as outcome variable. Due to a small numbers of observations, we did not include the covariates age, GDS and CIRS in the latter analysis. This study is part of a project where the main aim was to detect differences in one-year mortality between patients with hypoactive and hyperactive delirium, and we based power calculation on an assumption of 50% mortality among 60 patients with hypoactive delirium and 20% mortality among 40 patients with hyperactive delirium, giving a power of 87.9% with α = 0.05. We report odds ratios (OR) with 95% confidence intervals (CI) from the logistic regression analyses and judge two-sided *p*-values < 0.05 as statistically significant. We completed all analyses using SPSS version 25.

## Results

**Fig. 1:**
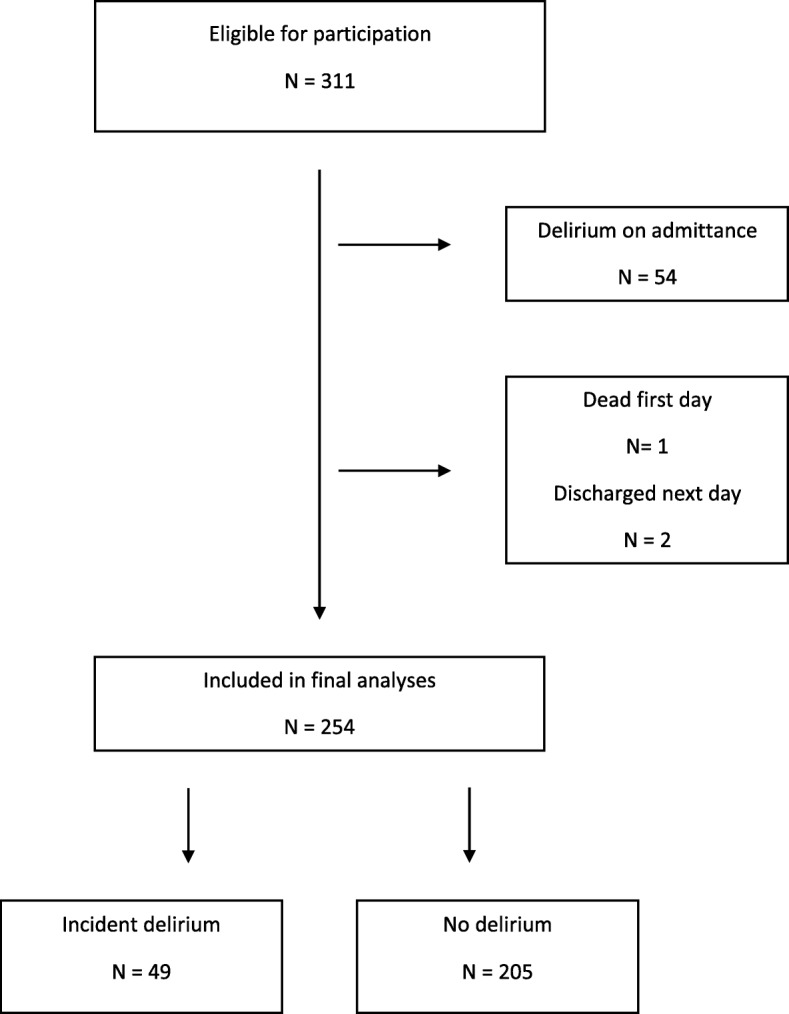
Flowchart

In total, 311 patients took part in the study. After reviewing the medical notes from the ED and other wards, we excluded 54 patients with delirium on admittance. As illustrated in Fig. 1 we excluded one patient who died the night of inclusion and two patients who were discharged the next day. This article thus reports analyses of 254 patients. As shown in Table 1, mean age was 86.1 years (SD 5.2), 151 (58.4%) were female and 133 (52.4%) had dementia.

**Table 1:** Baseline characteristics for all patients, patients with incident delirium and patients remaining free of delirium

	All (254) Mean; SD	Incident delirium (*n* = 49) Mean; SD	No delirium (*n* = 205) Mean; SD	*p*-values^a^
Age (years)	86.1; 5.2	86.9; 5.0	85.8; 5.2	0.20
Body Mass Index	24.2; 4.3	23.4; 3.6	24.4; 4.4	0.12
GDS^b^ (1–7)	3.4; 1.7	4.3; 1.3	3.2: 1.7	< 0.001
CIRS^c^ (0–56)	12.9; 4.4	14.3; 4.6	12.6; 4.3	0.020
APACHE (0–71)	8.9; 2.7	9.3; 2.7	8.8; 2.7	0.30
Barthel Index (0–20)	16.3; 3.6	14.9; 4.0	16.7; 3.4	0.002
SPPB^d^ (0–12)	4.0; 3.0	2.5; 2.6	4.3; 3.1	< 0.001
Female	151 (59.4%)	23 (46.9%)	128 (62.4%)	0.049
Home-dwelling	246 (96.9%)	46 (93.9%)	200 (97.6%)	0.19
Dementia (GDS > 4)	133 (52.4%)	38 (77.6%)	95 (46.3%)	< 0.001

Baseline characteristics for all patients, patients with incident delirium and patients remaining free of delirium

^b^Global Deterioration Scale

^c^Cumulative Illness Rating Scale

^d^Short Physical Performance Battery

Fourty-nine patients had incident delirium, of which we diagnosed 41 through direct assessment by the first author and the remaining eight through chart review. Eleven had hyperactive delirium, 11 mixed delirium, 18 hypoactive delirium and nine had no-subtype delirium. Thus, 22 patients had delirium with hyperactive symptoms and 27 had delirium without hyperactive symptoms. Regarding exposure variables, 42 out of 254 patients (16.5%) were transferred from other wards, 44 (17.3%) arrived at nighttime and 77 (30.3%) received nighttime investigations of which three had cerebral MRI-scans, 65 other radiological investigations and nine received visits from other specialities. Those arriving at nighttime received 25 (32.5%) of the nighttime investigations. The mean time spent in ED was 4.1 h (SD 0.9).

**Fig. 2:**
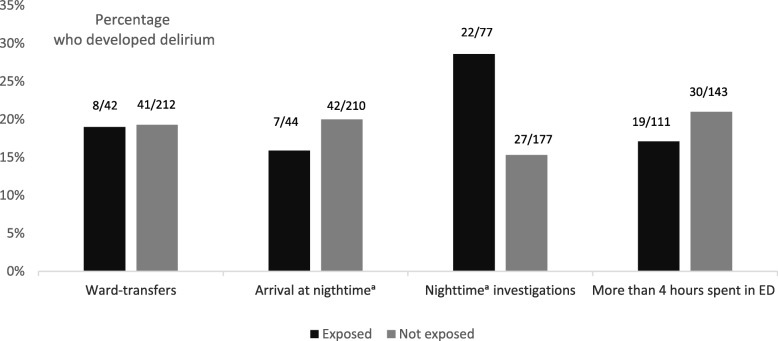
Percentages of patients who developed delirium among those who were exposed (black bars) and unexposed (grey bars) to the environmental factors. ^a^Between 8 p.m. and 8 a.m

Table 2 and Table 3 show results of the logistic regression analyses. In the unadjusted model, nighttime investigations were significantly associated with incident delirium (OR 2.22, CI 1.17 to 4.22, *p* = 0.015), indicating a more than doubled risk of incident delirium if the patient was exposed to nighttime investigations. In the multiadjusted model, nighttime investigations remained significantly associated with incident delirium (OR 2.61, CI 1.26 to 5.40, *p* = 0.010). Figure 2 illustrates the associations between ward-transfers, arriving at nighttime, nighttime investigations, time spent in ED and incident delirium. There were no significant associations between any of the exposure variables and the two groups of delirium motor subtypes.

**Table 2:** Logistic regression analyses with incident delirium (*n* = 49) as outcome variable, unadjusted and multiadjusted, for all the listed covariates, for all 254 patients

	Unadjusted			Multiadjusted		
	OR	95% CI	*p*-value	OR	95% CI	*p*-value
Ward-transfers	0.98	0.42 to 2.28	0.97	0.70	0.28 to 1.72	0.43
Arrive late^a^	0.76	0.32 to 1.82	0.53	0.56	0.20 to 1.55	0.26
Time spent in ED (hours)	0.89	0.75 to 1.06	0.20	0.85	0.69 to 1.04	0.12
Investigations at nighttime^a^	2.22	1.17 to 4.22	0.015	2.61	1.26 to 5.40	0.010
Age (years)	1.04	0.98 to 1.11	0.20	1.03	0.97 to 1.10	0.37
GDS^b^	1.54	1.24 to 1.91	< 0.001	1.59	1.26 to 1.99	< 0.001
CIRS^c^	1.09	1.01 to 1.17	0.020	1.08	1.00 to 1.18	0.049

^a^Between 8 p.m. and 8 a.m

^b^Global Deterioration Scale

^c^Cumulative Illness Rating Scale

**Table 3:** Logistic regression analyses with delirium with hyperactive symptoms (*n*=22) as outcome variable, unadjusted and multiadjusted, for all the listed covariates, for all 254 patients

	Unadjusted			Multiadjusted		
	OR	95% CI	*p*-value	OR	95% CI	*p*-value
Ward-transfers	1.14	0.36 to 3.54	0.83	1.11	0.35 to 3.49	0.86
Arrive late^a^	0.74	0.21 to 2.60	0.63	0.41	0.10 to 1.62	0.20
Time spent in ED (hours)	0.80	0.60 to 1.05	0.11	0.75	0.56 to 1.02	0.065
Investigations at nighttime^a^	1.67	0.68 to 4.09	0.26	2.00	0.77 to 5.16	0.15

^a^Between 8 p.m. and 8 a.m

## Discussion

In this observational study in acutely admitted geriatric patients, there was a significantly increased risk of incident delirium associated with exposure to nighttime investigations, even after adjusting for age, cognitive impairment and comorbidity, all well-known risk factors for delirium. There were no significant associations between ward-transfers, arrival at nighttime, time spent in ED and incident delirium. There were no associations between any of the exposure variables and delirium motor subtypes.

Previous studies have identified associations between room-transfers [15, 17] and time spent in ED [18] and delirium. These previous findings as well as our result seem biologically plausible since both room-transfers, long time spent in ED and nighttime investigations are potentially stressfull events that could induce aberrant stress responses which is a widely held hypothesis [19] regarding the pathophysiology of delirium. On the other hand, the association between nighttime investigations and incident delirium might be a spurious finding since the medical condition leading to nighttime investigations might be the true trigger of delirium, and not the investigation itself. The uncertainty about what is the true delirium trigger could have been reduced if we had reliable admission diagnoses, but in our opinion diagnoses on admission forms, at least in our hospital, are too unreliable to be used for this purpose. Like all observational studies, our study is unable to establish firm causality, and our findings must not be overinterpreted. In our opinion, clinicians must still refer geriatric patients to medically indicated nighttime investigations, but hospital organizers should secure that non-emergency investigations are done in a predictable way at daytime.

These associations between environmental factors and delirium are supported by studies showing that non-pharmacological, mainly environmental intervention programs are effective in preventing delirium [13, 14, 33]. Since delirium is common [1], has poor prognosis [3] and substantial economical impact [2], there seems to be a large potential for both health-related and economical benefits through focus on hospital environment and implementation of non-pharmacological delirium intervention programs. Such interventions also seem to have benefits beyond delirium prevention. In addition to a 44% reduction in delirium incidence, a meta-analysis from 2015 reports a 64% reduction in fall rates and a trend towards reduced length of stay and institutionalization rates in the intervention groups [13].

We found no association between ward-transfers and incident delirium. A possible explanation is that all ward-transfers are done in a predictable way at daytime. Another explanation may be that the geriatric ward provides a multicomponent intervention program against delirium that may out-weigh the potentially negative effect of ward-transfers. If so, this effect could also have influenced the associations between the other exposure variables and incident delirium. The lack of association between time spent in the ED and incident delirium might reflect that the ED in our hospital emphasises initial examination and short stay before transfer to a relevant ward, thereby providing less insult to vulnerable patients than the ED as described by Bo et al. [18]. Another explanation may be that physicians identify the patients prone to develop delirium as more vulnerable and examine these patients rapidly.

The lack of significant associations between any of the variables and delirium motor subtypes must be interpreted carefully due to a small number of observations. There is a trend towards less delirium with hyperactive symptoms with increasing time spent in the ED, which is plausible since the staff in ED might register signs of hyperactivity and therefore transfer these patients fast. An alternative hypothesis is that too quick transfers could be stressful and thereby inducing delirium. It remains uncertain if this trend would have reached statistical significance if the study was designed for this purpose. The results of previous studies addressing the relation between motor subtypes and etiology are diverging. A recent cross-sectional study found a negative association between use of atypical antipsychotics and hypoactive delirium and a positive association between intravenous lines and mixed delirium [11], possibly indicating differences in etiology between the subtypes. On the other hand, two reports from a longitudinal study designed to investigate the relationship between motor subtypes and other factors found no associations between motor subtypes and etiology [8], age and preexisting dementia [10]. Our results complies with the two latter reports.

### Strengths and limitations

The major strength of this study is that we have diagnosed delirium using the DSM-5 criteria directly and based the diagnoses on a combination of interviews with patients, nurses and proxies and a validated chart review method. The completeness of all variables of interest is another strength. The major limitation is the small number of patients with incident delirium. The limited sample size is particularly important when it comes to the analyses of environmental factors and delirium motor subtypes. Uncertainty about what is triggering delirium could have been reduced if we had reliable diagnoses for admissions to both hospital and nighttime investigations, and lack of such information is a limitation. A further limitation is that we were not strictly blinded to the exposure of environmental factors when diagnosing delirium, but we believe this has minor implications since we were focusing the presence of physiological disturbance resulting in delirium. Finally, our findings are not necessarily generalizable to non-geriatric wards and younger patients, or to EDs organized in a different way than in our hospital.

## Conclusions

In this observational study on 254 acutely admitted geriatric patients we found an association between nighttime investigations and incident delirium, but no associations between any of the exposure variables and delirium motor subtypes. In general, investigations should therefore be done in a predictable way at daytime, althoug patients should have emergency investigations at nighttime when indicated. Hospital environment in broad sense may be a target for delirium prevention along with non-pharmacological delirium intervention programs. There is a need for larger studies with both accurate registrations of environmental factors and a precise diagnostic work-up of delirium.

## Abbreviations

APACHE: Acute Physiology And Chronic Health Evaluation
BI: Barthel Index
CI: Confidence Interval
CIRS: Cumulative Illness Rating Scale
DMSS: Delirium Motor Subtype Scale
DSM: Diagnostic and Statistical Manual of Mental Disorders
ED: Emergency Department
GDS: Global Deterioration Scale
OR: Odds Ratio
SD: Standard Deviations
SPPB: Short Physical Performance Battery
